# A novel genetic technique in *Plasmodium berghei* allows liver stage analysis of genes required for mosquito stage development and demonstrates that *de novo* heme synthesis is essential for liver stage development in the malaria parasite

**DOI:** 10.1371/journal.ppat.1006396

**Published:** 2017-06-15

**Authors:** Upeksha L. Rathnapala, Christopher D. Goodman, Geoffrey I. McFadden

**Affiliations:** School of BioSciences, University of Melbourne, Parkville, Victoria, Australia; Seattle Biomedical Research Institute, UNITED STATES

## Abstract

The combination of drug resistance, lack of an effective vaccine, and ongoing conflict and poverty means that malaria remains a major global health crisis. Understanding metabolic pathways at all parasite life stages is important in prioritising and targeting novel anti-parasitic compounds. The unusual heme synthesis pathway of the rodent malaria parasite, *Plasmodium berghei*, requires eight enzymes distributed across the mitochondrion, apicoplast and cytoplasm. Deletion of the ferrochelatase (FC) gene, the final enzyme in the pathway, confirms that heme synthesis is not essential in the red blood cell stages of the life cycle but is required to complete oocyst development in mosquitoes. The lethality of FC deletions in the mosquito stage makes it difficult to study the impact of these mutations in the subsequent liver stage. To overcome this, we combined locus-specific fluorophore expression with a genetic complementation approach to generate viable, heterozygous oocysts able to produce a mix of FC expressing and FC deficient sporozoites. These sporozoites show normal motility and can invade liver cells, where FC deficient parasites can be distinguished by fluorescence and phenotyped. Parasites lacking FC exhibit a severe growth defect within liver cells, with development failure detectable in the early to mid stages of liver development *in vitro*. FC deficient parasites could not complete liver stage development *in vitro* nor infect naïve mice, confirming liver stage arrest. These results validate the heme pathway as a potential target for prophylactic drugs targeting liver stage parasites. In addition, we demonstrate that our simple genetic approach can extend the phenotyping window beyond the insect stages, opening considerable scope for straightforward reverse genetic analysis of genes that are dispensable in blood stages but essential for completing mosquito development.

## Author summary

Combating, and ultimately eliminating, malaria requires drugs that hit the parasite at all stages of its life cycle. Deletions of parasite metabolism genes can expose where and when a certain metabolic pathway is essential, and hence vulnerable to drugs. However, once a parasite gene has been deleted, it is difficult to examine that parasite beyond the point in the life cycle where that gene is essential because the parasites die. We developed a simple genetic complementation strategy to bridge malaria parasites through the mosquito life stage when they carry a genetic defect that otherwise specifically stops them completing this stage. Our strategy allows the phenotypic analysis of any such defect in the subsequent life stages, in any genetically tractable parasite species. It does not require specific background lines, or the chemical or physical treatment necessary for conditional knockout approaches and can be applied to any of the ~50 genes already known to be specifically required for development in the mosquito. This includes genes of the heme synthesis pathway. Malaria parasites are obliged to synthesize the iron-managing molecule heme to complete their development in mosquitoes. We used our genetic complementation strategy to bridge malaria parasites lacking the final enzyme in the heme synthesis pathway through the mosquito phase and back into a vertebrate. We discovered that parasite-generated heme is crucial for the parasite to complete the liver phase of vertebrate development; absence of this enzyme prevents parasites from progressing to the symptomatic blood phase. Our results indicate that blocking parasite heme production should work as a prophylactic intervention to prevent the progression of incipient malaria infections into the deadly blood phase.

## Introduction

Malaria is a life-threatening disease caused by five species of *Plasmodium* parasites, with *P*. *falciparum* being the most deadly. The on-going emergence of drug resistance threatens malaria control efforts [1] and highlights the need for new drugs and novel approaches for controlling the spread of this disease. *Plasmodium berghei* is a widely-used mouse malaria model and a dominant tool for reverse genetic studies in malaria [2]. The *P*. *berghei* genome is largely homologous to the genome of the human parasites, which permits experiments that are difficult to perform with human subjects [3]. Experimental access to the entire life cycle is arguably the most important advantage of rodent malaria parasites as it facilitates study of transmission dynamics, exoerythrocytic stage vaccines, and prophylactic drugs.

Genome manipulation is an essential tool for understanding key events in the *Plasmodium* life cycle. Stable transfection of the parasite and modification of its genome by homologous recombination are now common procedures [4]. A significant limitation of the method is that the parasite genes essential at one life stage cannot be characterized in subsequent stages. In the growing list of loss of function mutations that can survive the blood stages, many are found to fail during development in the mosquito; possibly due to the increased metabolic requirements in this stage of the life cycle [5]. Failure to produce infective sporozoites limits the possibilities for studying the role of these genes in the liver stage. Given the unique features of the hepatic stage of the life cycle, particularly the low parasite burden and the role in generation of sterile immunity [6], understanding parasite biology at this stage is of great importance. Thus, temporal gene inactivation is needed in functional studies to test protein function during liver stage development. A small number of genes essential in other life stages have been successfully ablated using conditional knockout strategies in *P*. *berghei* to analyse liver stage function [7, 8], and other conditional strategies are available. But these strategies can present significant limitations in terms of which species can be tested, the complexity of plasmid construction, the need for specific parasite lines, requirements for specific chemical or physical treatments during the life cycle, and unpredictable variations in the effectiveness of the gene ablation. Such drawbacks have limited the use of these techniques and severely hampered analysis of parasite liver stage responses to gene deletion [9, 10] largely precluding reverse genetic approaches to this important life cycle phase.

Heme is essential in malaria parasites, playing an important role as a precursor for cytochrome production in the mitochondrial electron transport chain. *Plasmodium* parasites encode and express all the enzymes for a complete heme biosynthesis pathway, and heme biosynthesis was initially considered a potential drug target against malaria parasites [11]. However, the *de novo* heme synthesis pathway enzymes are dispensable during the blood stage of the parasite life cycle [12–15]. It appears that parasites still require heme, but in the blood stage they can scavenge pathway intermediates, heme and/or heme synthesizing enzymes, from the host red blood cell [12, 14, 16]. However, these alternate sources are apparently not sufficient or accessible enough during the mosquito stages of the parasite life cycle, where parasites lacking heme-synthesizing enzymes fail to complete sporozoite development in the oocyst [12, 13, 15].

Given the essentiality of the endogenous heme biosynthesis pathway in the mosquito stages of the parasite life cycle, it is hardly surprising that there is minimal data available about the liver stage growth of heme deficient parasites. Parasites lacking the first enzyme in the heme biosynthesis pathway, aminolevulinic acid synthase (ALAS) can only complete the life cycle if mosquitoes are fed aminolevulinic acid (d-ALA), the product of ALAS [12, 15]. This chemical complementation approach has been effectively employed to generate infective ALAS-minus sporozoites and demonstrate that parasites can scavenge d-ALA from the host liver and, thereby, partially restore parasite viability [15]. While it is clear that parasites can survive the loss of ALAS, this finding does not directly address whether parasite synthesized heme is essential for liver stage development. Aminolevulinic acid is a stable compound that appears to be available for uptake from the host cell cytoplasm, possibly by the same mechanism that imports parasite synthesized d-ALA into the apicoplast where the next steps in the pathway are localized. It remains unclear if the other compensatory mechanisms available to parasites in the red blood cell, particularly the ability to scavenge FC and/or heme, are available to the parasite during liver stage infection.

A chemical complementation approach is not available for the final stage of the heme biosynthesis pathway, namely conversion of protoporphyrin IX and iron to heme by ferrochelatase (FC) in the mitochondrion, so we set out here to address this limitation by applying a genetic complementation method to rescue parasites with a deletion of the FC gene. By combining locus-specific fluorescence tagging with the ability of sexual outcrossing to bridge parasites through the mosquito life stages [17], we generated viable sporozoites that lacked the FC gene but were capable of infecting liver cells *in vitro* and *in vivo*. The specific fluorescent labelling of parasites lacking FC allowed detailed exploration of the reliance on heme for parasite development in the liver stage. Our fluorescence-tagging complementation strategy gives a straightforward method for reverse genetic analysis of various blood-stage dispensable/mosquito-stage essential genes that is useable in all genetically tractable, fertile *Plasmodium* species and requires no special conditions.

## Materials and methods

### Experimental animals

Male asmu:Swiss mice aged between 4–6 weeks obtained from Monash Animal Research platform were used in all the experiments.

### Ethics statement

All animal experiments were carried out as per the National Health and Medical Research Council—Australian Code for the Care and Use of Animals for Scientific Purposes 2013 (8^th^ edition) guidelines and were permitted by the University of Melbourne Animal Ethics committee under Ethics ID 1413078.1. Anesthesia using ketamine/xylazine, euthanasia via slow fill carbon dioxide followed by cervical dislocation.

### Parasites

The *Pb*nek-4ko strain [18] was provided by Oliver Billker (Wellcome Trust Sanger Institute, Hinxton, Cambridge, UK). *Pb ANKA* parasites lines expressing GFP, tdTomato and mCherry fluorescence markers were developed in our laboratory based on [19]. See Supplementary Methods for a full description of the generation of these lines.

### Generation of *Plasmodium berghei* ^FC^ knockout parasites

To generate the *P*. *berghei* FC (Pb ANKA_114070) knockout parasites (FC^KOmCh^), the selection cassette from pLChSKD, a modified version of pL0006 (MRA-775) with the hDHFR replaced by mCherry fused to hDHFR via a viral 2A skip peptide [20], was ligated into the *Xho*I/*Bgl*II sites of pL0006 containing regions flanking the FC gene [16] to create pFCChSKD. See Supplementary Methods for a full description of pLChSKD plasmid construction. *Pb*ANKA parasites were transfected with *Hind*III/*Eco*RI linearized pFCChSKD, genotyped by PCR (see S1 Table for primer sequences) and cloned as previously described [5, 21].

### Infection of *Anopheles stephensi*

All parasite strains used for coinfection crossing experiments were inoculated by intraperitoneal injection into naïve mice and assessed for fluorescence and genotype at day 3. 2.5 x 10^5^ parasites from each strain were mixed and used to infect a naïve mouse by intravenous (IV) injection. The mice were infected with the following combinations of *P*. *berghei* parasitic strains.

1. FC^KOmCh^ x FC^WT^-GFP–Parasites having FC replaced with ChSKDHFR mixed with parasites having a wild type FC locus and expressing GFP from a different genetic locus
2. FC^KOmCh^ nek-4wt x FC^WT^-nek-4ko—Parasites with a wild type nek4 locus and having FC replaced with ChSKDHFR mixed with parasites carrying a wild type FC gene but lacking nek-4.
3. FC^WT^-mCh x FC^WT^-GFP–parasites with a wild type FC locus and expressing mCherry from another genetic locus mixed with FC wild type parasites expressing GFP from another genetic locus.
4. FC^WT-^mCh x FC^WT^-nek-4ko—parasites with wild type FC and nek4 loci and expressing mCherry from another genetic locus mixed with non-fluorescent parasites with a wild type FC locus but lacking nek-4.

Adult females of *A*. *stephensi* (MR4) reared under standard insectary conditions (adult mosquitoes were grown at 27°C with humidity of 80% and light: dark photoperiod of 14:10 hours with a 1 hour ramp in) were allowed to feed on 4–6 weeks old Swiss mice anesthetized with ketamine/xylazine 3 days following coinfection. A minimum of three independent infections were done for all crosses.

### Assessment of mosquito stage development

Twelve days after infection, mosquito midguts were removed, imaged using a Leica DM 2500 epifluorescence microscope (20x) and counted. Oocyst size was measured using Fiji (ImageJ 1.46a, Wayne Rasband, National Institutes of Health).

Salivary gland sporozoite numbers were counted using a standard haemocytometer and averaged per mosquito. Salivary gland sporozoite motility 21 days post-infection was analysed by immunofluorescence assay (IFA) [22]. Slides were probed with primary antibodies against anti-circumsporozoite protein (1: 500; a gift from Louis Schofield, Walter and Eliza Hall Institute) and mCherry (1: 250; Abcam ab167453). FC^KOmCh^ nek-4wt x FC^WT^-nek-4ko parasites were probed with Alexa Fluor 488 goat anti-mouse IgG and Alexa Fluor 546 goat anti-rabbit IgG (1:1000; Molecular Probes) secondary antibodies. FC^KOmCh^ x FC^WT^-GFP parasites were probed with Alexa Fluor 633 goat anti-mouse IgG and Alexa Fluor 546 goat anti-rabbit IgG (1:1000; Molecular Probes/ThermoFisher) secondary antibodies. Salivary gland sporozoite gDNA was extracted and used as template for nested PCR to confirm the presence of FC^KOmCh^ parasites.

### *In vitro* liver stage development analysis

Growth and IFAs of pre-erythrocytic forms were performed as described [23]. To determine size and nuclear content, parasites were stained with rabbit anti-mCherry (1: 250; Abcam ab167453)/Alexa Fluor 546 goat anti-rabbit IgG (1:1000; Molecular Probes/ThermoFisher) and Hoechst 33342 (5μg/ ml). Images collected at 40x magnification with the Leica DM 2500 microscope were analysed in Fiji to measure parasite size in μm^2^. Student t-test (unpaired) was used to compare the average growth size differences 24, 48 and 68 hours post infection for parasites pooled from 6 independent experiments. For nuclear content, Chi-squared analysis was used to assess pooled data from two independent experiments.

To further assess parasites development in liver cells, parasites grown for 68 hours in three independent experiments were stained with both rabbit anti-mCherry, (1: 250; Abcam ab167453) and mouse anti-Pf merozoite surface protein-1 (MSP-1) (1: 100; a gift from Paul Gilson, Burnet Inst) followed by Alexa Fluor 546 goat anti-rabbit IgG Probes and Alexa Fluor 488 goat anti-mouse IgG (1:1000; Molecular Probes/ThermoFisher) for the FC^KOmCh^-nek4-wt x FC^WT^-nek4-ko. For the FC^KOmCh^ x FC^WT^-GFP cross, Alexa Fluor 633 goat anti-mouse IgG and Alexa Fluor 546 goat anti-rabbit IgG (1:1000; Molecular Probes/ThermoFisher) were used as secondary antibodies. MSP-1 expression was compared using Fisher’s exact test of data pooled from three independent experiments (801 parasites assessed).

To determine the ability of parasites to complete liver stage development *in vitro*, merosomes were counted at 70 hpi using an Olympus CKX41 compound fluorescence microscope at 40x magnification.

To confirm the presence of FC knockout parasites in liver by PCR, naïve mice were (IV) infected with 20,000 sporozoites. Forty to 42 hours post infection, mice were sacrificed, their livers harvested and genomic DNA was extracted for use as template in a nested PCR reaction. See S1 Table/S1 Fig for primer sequences and PCR strategy.

### Analysis of sporozoite infectivity and ability to infect naïve mice

For bite-back infection, mosquitoes were given access to anesthetized Swiss mice (4–6 weeks old) until at least 20 mosquitoes had fed. For IV infection, salivary gland sporozoites were isolated from salivary glands 21–24 days after infection, counted and IV injected at 1000 or 10,000 sporozoites/mouse. Time to patency and the subsequent development of asexual stage parasites, were monitored by fluorescence microscopy and Giemsa stained blood smears from 3 days post infection onwards. Genotyping by PCR was done as described above.

For each pyrimethamine selection of FC^KOmCh^ parasites, two mice were simultaneously infected from a single cage of mosquitoes. One mouse was given pyrimethamine in its drinking water (70μg/ ml) while the other was not treated. Once parasites were detected in the untreated mice, they were analysed for fluorescence and genotype and then given pyrimethamine in drinking water (70μg/ ml) to clear all the parasites. After parasites were cleared (day 4) pyrimethamine was removed and mice were observed for 7 days to test for recrudescence.

### Statistical analysis

All statistical analysis was done using GraphPad Prism (GraphPad Software, La Jolla California USA)

## Results

### Deletion of the ferrochelatase gene arrests parasite development in the oocysts stage

*Pb* ANKA_114070 encodes FC, the last enzyme of the heme biosynthesis pathway [24]. We interrupted the *Pb* ANKA_114070 locus by double cross over homologous recombination introducing the selectable marker, human dihydrofolate reductase (*h*DHFR) fused to a fluorescent mCherry tag and driven by the constitutive *Pb* EF-1α promoter, into the FC coding sequence (S1 Fig). Parasites carrying this FC deletion are denoted as FC^KOmCh^. When *Anopheles stephensi* mosquitoes fed on mice infected with FC^KOmCh^, oocysts developed in these mosquitoes but no salivary gland sporozoites were observed (Table 1). This is consistent with previous reports that FC is dispensable at the blood stage and early sexual stages but crucial for completion of sporozoite development [12, 13, 16].

**Table 1:** Life cycle progression and infectivity of FC^KOmCh^ parasites following outcrossing.

Parasite lines	Number of infections	Infection rate (%)/ Oocysts per mosquito* (n = number of infected mosquitoes)	Sporozoites per mosquito (n = number of mosquitoes dissected)	Transmission to naïve mice**		Time to patency (days) **
				IV injection (n = number of sporozoites injected)	bite-back	
**FC**^**KOmCh**^ **parent**	2	47 ± 29/ 203 ± 34 (n = 13)	0 n = 22	(NA)***	0/1	N/A
**FC**^**KOmCh**^ **x FC**^**WT**^**-GFP**	4	79 ± 5/ 172 ± 30 (n = 33)	6367± 1206 n = 72	n = 1000–1/1 n = 10 000–1/1	4/4	4.5
**FC**^**WT**^**-mCh x FC**^**WT**^**-GFP**	3	80 ± 2/ 161 ± 33 (n = 24)	6101± 233 n = 78	(ND)	3/3	5
**FC**^**KOmCh**^**nek-4wt x FC**^**WT**^**-nek-4ko**	8	86 ± 4/ 181 ± 21 (n = 75)	5330± 906 n = 142	n = 1000–1/1 n = 10 000–1/1	4/4	4.75
**FC**^**WT**^**-mCh-nek-4wt x FC**^**WT**^**-nek-4ko**	5	79 ± 6/ 241± 28 (n = 43)	10913± 3482 n = 94	n = 10 000–1/1	3/3	4.5

*The number of oocysts per midgut reflect only mCherry expressing oocysts from infected mosquitoes

**Transmission and days to patency data do not include results of experiments involving pyrimethamine treatment.

***Pb FC^KOmCh^ parasites do not produce salivary gland sporozoites when self-fertilized, so IV injection was not possible.

NA = not applicable. ND = Not done.

### Crossing ferrochelatase deficient parasites with those carrying a wild type ferrochelatase gene complements the defect in oocyst development and sporozoite production

**Fig 1:**
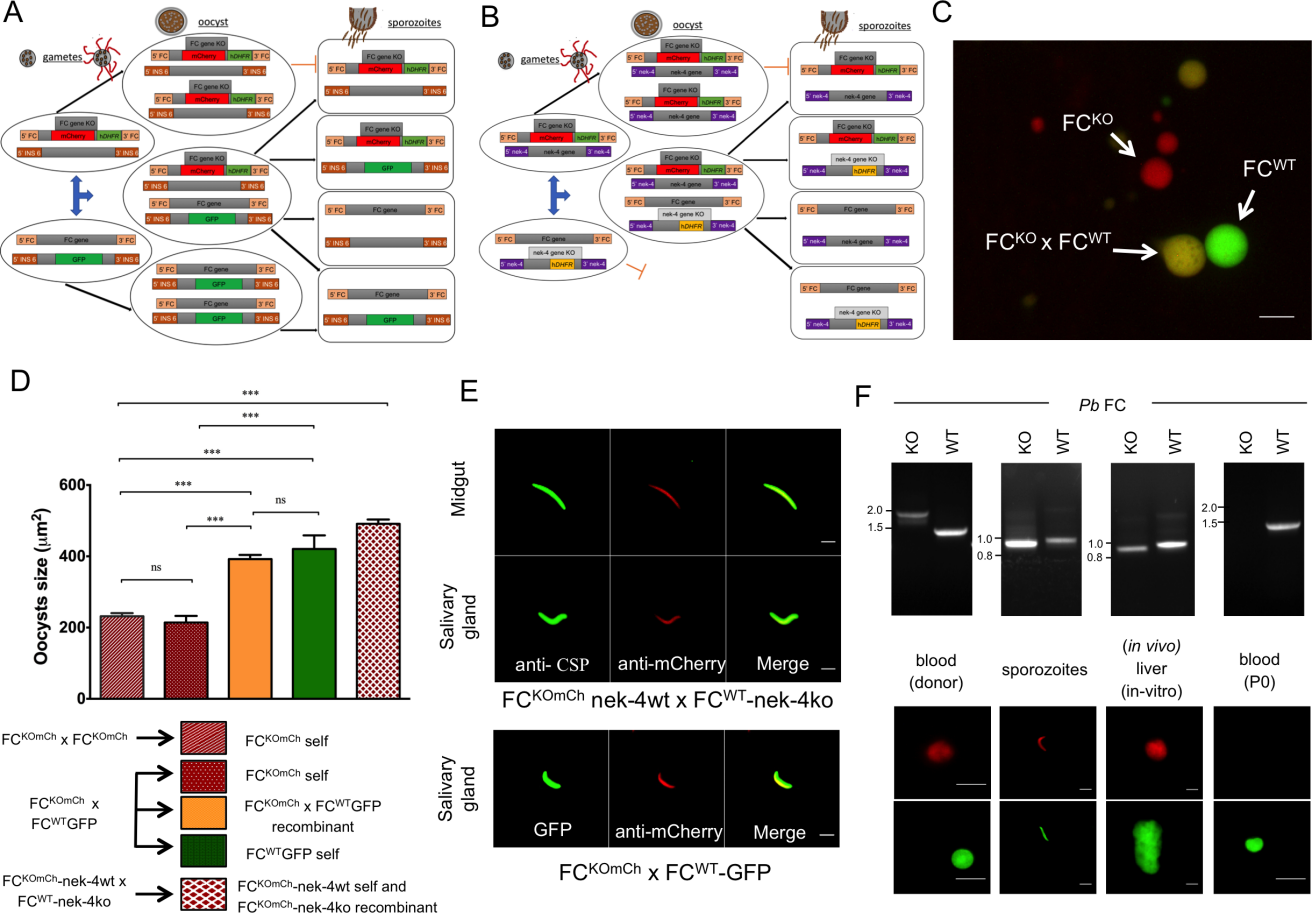
Crossing FC^KOmCh^ to FC^WT^ parasites allows normal development of FC^KOmCh^ sporozoites. A) Schematic representation of the predicted parasite genotypes at the polyploid oocyst stage and haploid sporozoite stage after allowing mosquitoes to feed on mice dually infected with FC^KOmCh^ and FC^WT^-GFP parasites. Three genetic combinations are possible in the polyploid oocyst: self- fertilization yielding oocysts with only the FC^KOmCh^ or the FC^WT^-GFP allele or outcrossing creating an oocyst with both FC^KOmCh^ and the FC^WT^-GFP alleles. Following sporogony, the parasite returns to the haploid state yielding four possible genetic combinations of the FC^KOmCh^ and the FC^WT^-GFP genes: The original FC^KOmCh^ or FC^WT^-GFP alleles alone, a combination of both the FC^KOmCh^ and FC^WT^-GFP alleles or parasites carrying neither allele. B) Schematic representation of the predicted parasite genotypes at the polyploid oocyst stage and haploid sporozoite stage after allowing mosquitoes to feed on mice dually infected with FC^KOmCh^–nek4-wt and FC^WT^-nek4-ko parasites. The nek4ko mutation prevents self-fertilization of this parasite line, so two genetic combinations are possible in the polyploid oocyst: self- fertilization yielding oocysts with only the FC^KOmCh^ allele or outcrossing creating an oocyst with both FC^KOmCh^ and the FC^WT^-nek4ko alleles. Following sporogony, the parasite returns to the haploid state yielding four possible genetic combinations of the FC^KOmCh^ and the FC^WT^-nek4ko genes: The original FC^KOmCh^ or FC^WT^-nek4ko alleles alone, a combination of both the FC^KOmCh^ and FC^WT^-nek4ko alleles or parasites carrying neither allele. C) A midgut infected by feeding on an FC^KOmCh^ x FC^WT^-GFP infected mouse showing the presence of the three oocyst phenotypes. Red oocysts are self-fertilized FC^KOmCh^ parasites, green are self-fertilized FC^WT^-GFP, and orange oocysts are FC^KOmCh^ x FC^WT^-GFP recombinants. scale bar: 20μm. (D) Complementation of the FC^KO^ phenotype demonstrated by comparing the mean surface area of oocysts from FC^KOmCh^ self-fertilization with oocysts produced by the FC^KOmCh^ x FC^WT^-GFP and FC^KOmCh^ nek-4wt x FC^WT^-nek-4ko crosses. The female specific defect resulting from the nek4-ko genotype prevents development of colourless FC^WT^-nek-4ko oocysts, so all oocysts produced from FC^KOmCh^ nek-4wt x FC^WT^-nek-4ko crosses carry the FC^KOmCh^ and are red in colour. ns; not significant, ***—*P* value <0.0001 (Student t test) (E) Immunofluorescence assay showing the presence of mCherry fluorescence, and hence the presence of the FC^KOmCh^ allele in midgut (day 17 post infection) and salivary gland (day 21 post infection) sporozoites from the FC^KOmCh^ nek-4wt x FC^WT^-nek-4ko cross and the salivary gland sporozoites from the FC^KOmCh^ x FC^WT^-GFP cross. scale bar: 10μm F) FC genotype (by PCR) and matching fluorescent phenotype of parasites through the complete life cycle showing the presence of parasites carrying both the FC^KOmCh^ and FC^WT^ genes until the liver stage of the life cycle. Parasites carrying the FC^KOmCh^ fail to complete liver stage development so neither red fluorescence nor the FC^KOmCh^ gene is detectable in the subsequent blood stage infection. Sporozoite and liver stage genotypes were assessed with a nested PCR reaction, yielding a band of different size to that seen in blood stages. scale bar: 10μm.

To determine if the effects of the FC deletion during the mosquito stage could be complemented by a wild type copy of the allele (denoted FC^WT^), we crossed our FC^KOmCh^ with two different strains carrying intact FC genes: either FC^WT^ parasites expressing GFP inserted into the intergenic locus of chromosome 6 [19], or a female sterile line FC^WT^-nek-4ko [18]. To control for any effects of fluorophore expression on oocyst generation and development, we also generated control crosses of the fluorescent lines with parasites with a wild type FC locus. Mice were dual infected and *A*. *stephensi* mosquitoes fed on these dual infected mice to generate the various crosses. Expected genotypes of the oocysts and sporozoites from the two crosses are depicted in Fig 1A and 1B.

The replacement of the FC coding region with *h*DHFR fused to mCherry in FC^KOmCh^ parasites allows tracking of FC^KO^ genotype throughout sexual reproduction. In mosquitoes fed on mice infected with both FC^KOmCh^ and FC^WT^-GFP, after sexual recombination and oocyst formation in mosquitoes, three oocyst genotypes could be identified 12 days post infection: FC^KOmCh^ (red), FC^WT^-GFP (green), and FC^KOmCh^ x FC^WT^-GFP recombinants (orange) (Fig 1C).

Mosquito infectivity, as measured by infection rate and oocyst load, was not significantly different (P>.05, one-way ANOVA) between FC^KOmCh^ and FC^WT^-mCh parasites when crossed to either FC^WT^-GFP or FC^WT^-nek-4ko parasites (Table 1). This confirms previous findings [12, 15] that the parasite heme synthesis enzymes do not play an essential role in the early mosquito life stages in *P*. *berghei*.

FC deletion restricts the growth of oocysts (Fig 1C and 1D). Fluorescence tags allowed us to determine the genotype (presence/absence of active FC gene) of each oocyst. In the FC^KOmCh^ x FC^WT^-GFP cross, oocysts with only mCherry fluorescence (red) are the product of FC^KOmCh^ self-fertilization, and they exhibit the reduced oocyst growth phenotype (Fig 1C and 1D) characteristic of parasites lacking ferrochelatase, or other heme synthesizing enzymes [15]. FC^KOmCh^ self-fertilized parasites (red in the cross) are not significantly different in size to oocysts produced when mosquitoes are infected with FC^KOmCh^ alone (Fig 1D). In contrast, orange oocysts, which carry both FC^KOmCh^ and FC^WT^-GFP genes, are significantly larger than exclusively FC^KO^ oocysts (red) and equivalent in size to FC^WT^-GFP oocysts (green), which are self-fertilized progeny of the GFP containingparent from FC^KOmCh^ x FC^WT^-GFP cross. A comparable increase in size was also seen in the oocysts from the FC^KOmCh^-nek-4wt x FC^WT^-mCh-nek-4ko cross. Because the FC^WT^-mCh-nek-4ko line is female sterile [18], no colourless oocysts were produced, and in the absence of a second fluorophore it is not possible to distinguish FC^KOmCh^ nek-4wt self-fertilization oocysts (red) from FC^KOmCh^ nek-4wt x FC^WT^-mCh-nek-4ko recombinants (red). Nevertheless, the mCherry expressing oocysts from the FC^KOmCh^ nek-4wt x FC^WT^-mCh-nek-4ko cross are, on average, significantly larger than oocysts arising from infections with FC^KOmCh^ alone (Fig 1D).

Complementation of the FC knockout by crossing to FC^WT^ strains not only restored normal oocyst growth but also enabled development of sporozoites, with robust numbers of salivary gland sporozoites produced in all our crosses (Table 1). Red sporozoites could be observed in the mosquito midgut at day 17 and in the salivary glands at day 21 post-infection (Fig 1E). Sporozoites contain only a single haploid nucleus, so red sporozoites predict the presence of FC^KOmCh^ genotype in the sporozoites generated by the cross and this was confirmed by PCR (Fig 1F). Therefore, we conclude that introduction of a wild type FC gene by sexual crossing can restore oocyst growth and sporozoite production to FC^KO^ parasites. We noted that all the sporozoites from the FC^KOmCh^ nek-4wt x FC^WT^-nek-4ko cross were red, despite the expectation that half of the sporozoite population should carry the colourless FC^WT^ genotype and, therefore, not fluoresce. The presence of both FC^KOmCh^ and FC^WT^ parasites was confirmed by PCR (Fig 1F). This suggests that fluorescent protein was being carried over from the oocyst and interfering with visual genotyping of sporozoites in the midgut and salivary glands.

**Fig 2:**
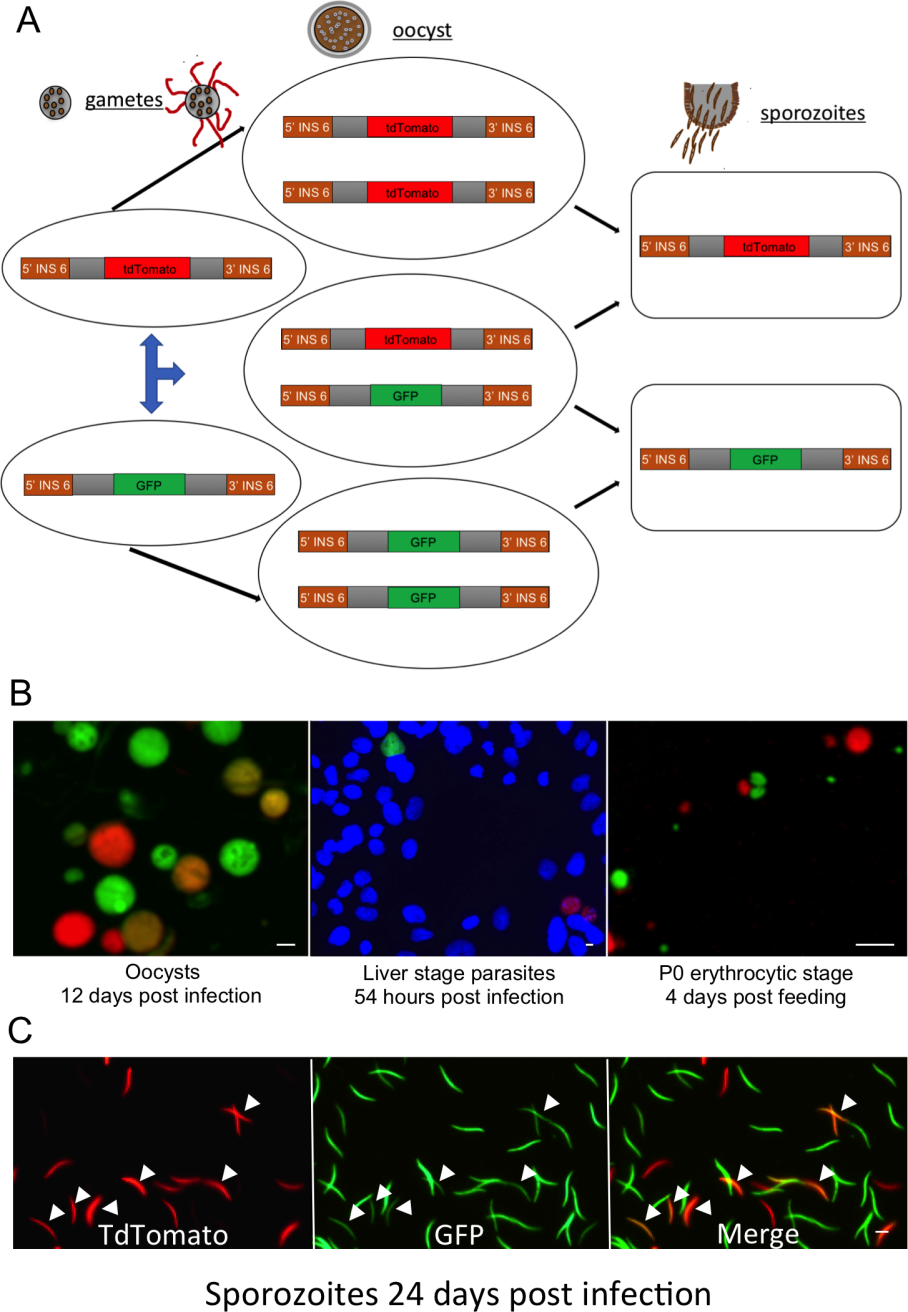
Sporozoite development involves significant carryover of oocyst cytoplasm. A) Schematic representation of the predicted parasite genotypes at the polyploid oocyst stage and haploid sporozoite stage after allowing mosquitoes to feed on mice dually infected with parasites expressing either GFP or tdTomato. The fluorophore is inserted into the same genetic locus in each parasite line, so three genetic combinations are possible in the polyploid oocyst: self- fertilization yielding oocysts with either the GFP or tdTomato allele, whereas outcrossing generates a heterzygous oocyst with both the GFP and tdTomato alleles. Following sporogony, the parasite returns to the haploid state and can carry either the GFP or tdTomato allele, but not both. B) Progeny of the GFP x tdTomato cross through the life cycle. Three oocyst phenotypes are present, GFP only (green) and tdTomato only (red) from self-fertilizations and heterozygous, polyploid parasites expressing both fluorophores (orange). Only two phenotypes, GFP (green) or tdTomato (red) are present in haploid liver and red blood cell stages. C) Sporozoites generated from crossing GFP and tdTomato parasites showed many haploid sporozoites still carrying both fluorphores (arrow heads). Single colour (green or red) sporozoites are presumably offspring from self-fetilizations, while two-colour (orange) sporozoites are from recombinant oocysts.

To further examine the apparent carryover of fluorescent protein from the oocyst to the sporozoites, we crossed parasites with either GFP or tdTomato inserted in the same genetic locus (Fig 2A), namely the intergenic region of chromosome 6 [19]. Oocysts from this cross exhibited three different colours (green, red and orange) indicating that the lines had successfully crossed and also self-fertilised (Fig 2B). Unlike the polyploid oocysts, haploid sporozoite progeny from this cross can inherit only one fluorophore gene, either GFP or tdTomato (Fig 2A). Nevertheless, in addition to exclusively green or red sporozoites, we observed many sporozoites that contained both fluorophores (orange parasites highlighted with arrows in Fig 2C). We conclude that sporozoite formation involves carryover of significant oocyst cytoplasm, resulting in dual-coloured sporozoites emerging from the heterozygous oocysts. All parasites had reverted to exclusively GFP or tdTomato once the sporozoites invaded liver cells and developed into early exoerythrocytic forms (Fig 2B). Similarly, only red or green parasites were observed when these parasites progressed in to blood stage following bite-back (Fig 2B)

### FC^KO^ sporozoites are liver infective both *in vitro* and *in vivo* but have severe growth defects and fail to complete liver stage development

To examine the viability of FC^KOmCh^ sporozoites, we tested their motility *in vitro*, and their infectivity both *in vitro* and *in vivo*. No significant difference between the number of motile sporozoites, or in their levels of motility, was observed between FC^KOmCh^ and FC^WT^ sporozoites (S2 Table, S2 Fig). FC^WT^ parasites had slightly more sporozoites with maximal motility (> 10 trails), but this difference was not statistically significant (P>0.05).

**Fig 3:**
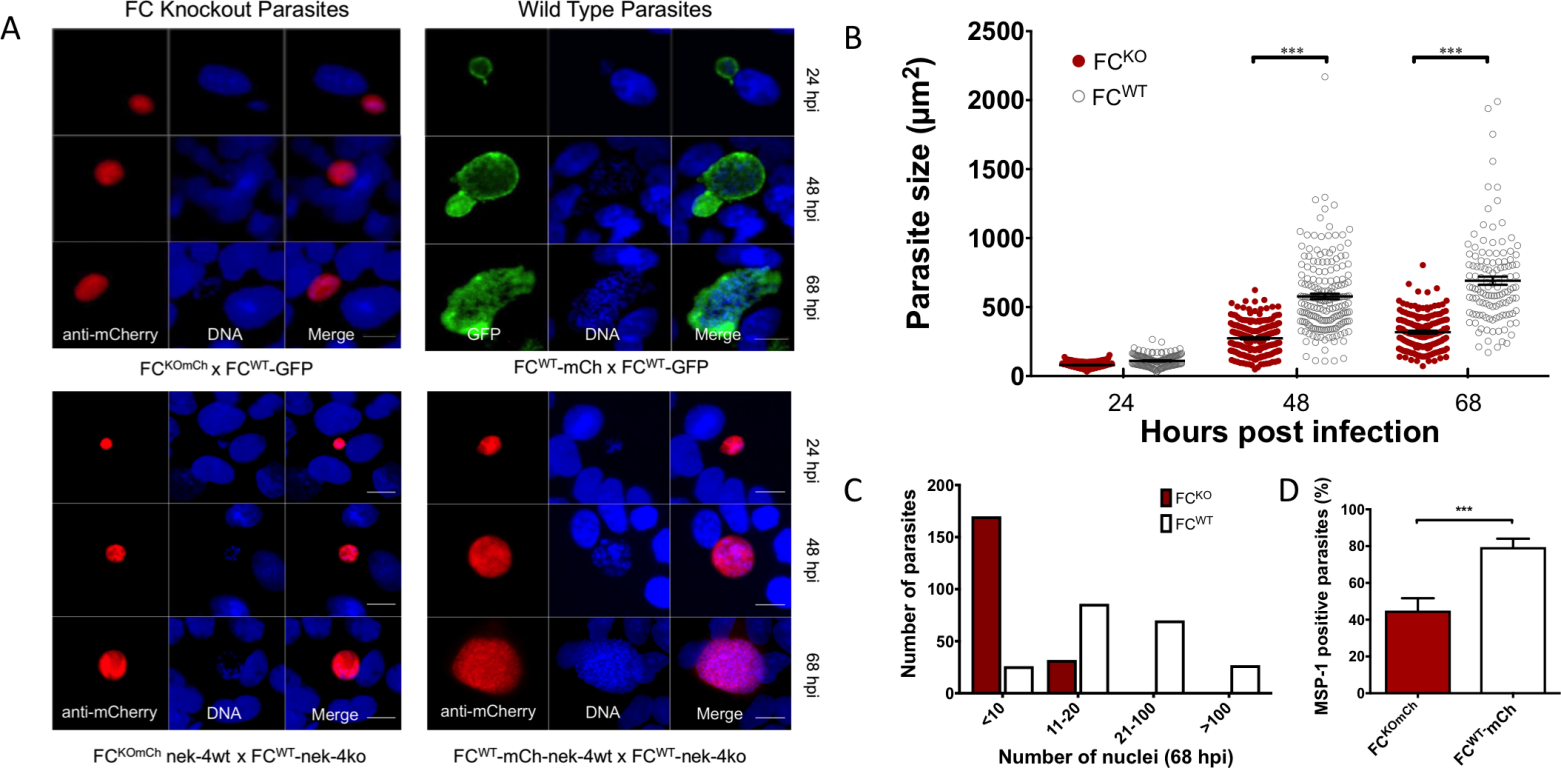
Parasites lacking FC have a severe liver stage growth phenotype. A) Immunofluorescence assay of FC^KOmCh^ and FC^WT^ parasites grown in HepG2 liver cells for 24, 48 and 68 hours post infection (hpi) showing the reduced growth of FC^KOmCh^ parasites. scale bar 20 μm. B) Quantification of liver stage parasite size showing that FC^KOmCh^ parasites are significantly smaller than FC^WT^ parasites after 48 and 68 hours of incubation in HepG2 cells. ***—P value < 0.0001 (Student t-test), data pooled from six independent experiments. C) Quantification of parasite nuclei 68 hours after infection showing that FC^KOmCh^ parasites produced significantly fewer nuclei than FC^WT^ parasites by 68 hpi. P value <0.0001 (χ^2^). D) Immunofluorescence assay of MSP-1 expression, a marker for mid to late stage parasite development in the liver stage. FC^KOmCh^ parasites have a defect in MSP-1 expression, with only 45% of FC^KOmCh^ parasites producing MSP-1 compared to 79% of FC^WT^. ***—P value <0.001(Fisher’s exact test) 801 parasites assessed, pooled from three independent experiments with no fewer than 58 parasites measured in any one sample. Error bars show 99% confidence interval.

Sporozoites recovered from complemented FC^KOmCh^ lines were used to infect *in vitro* cultures of HepG2 liver cells, and parasite development was assessed by IFA at 24, 48 and 68 hours. By 24 hours, any residual fluorescent protein seen in the sporozoites is lost and the liver parasites can be unequivocally genotyped by fluorescence expression, with mCherry expression denoting the FC^KOmCh^ genotype. FC^KOmCh^ liver parasites (red) could be detected at 24, 48 and even 68 hours post infection from either of the complementation crosses (Fig 3A). The FC^KOmCh^ (red) parasites were smaller than FC^WT^ parasites at all three time points, although the difference only becomes statistically significant at 48 and 68 hours (Fig 3A and 3B). This size defect correlated with a significant reduction (P<0.0001) in the number of nuclei produced in FC^KOmCh^ parasites at 68 hpi (Fig 3C). To further assess progress through liver stage development, the production of merozoite surface protein-1 (MSP-1) was measured in FC^KOmCh^ parasites. Significantly fewer FC^KOmCh^ parasites (45%) were MSP-1 positive than FC^WT^ type parasites (79%) (Fig 3D, P<0.001) suggesting a mid-stage liver stage defect [25–28].

To determine if the smaller size, reduced nuclear content, and impaired MSP-1 expression in FC^KOmCh^ liver stage parasites foreshadowed an inability to complete the liver stage of the life cycle we counted merosomes, which are groups of membrane-bound merozoites that bud off the infected liver cell and represent the end-product of the parasite’s liver stage development [29]. Merosomes were readily recovered, but none contained red fluorescent parasites, indicating that FC^KOmCh^ parasites could not complete the liver stage *in vitro* (Table 2). We conclude that FC^KOmCh^ parasites can infect liver cells *in vitro* but cannot complete liver stage development.

**Table 2:** Production of FC^KOmCh^ merosomes following outcrossing (no. of trials = 3).

	FC^KOmCh^	FC^WT^-mCh
70 hours post infection	0	21.6 ± 6

**Fig 4:**
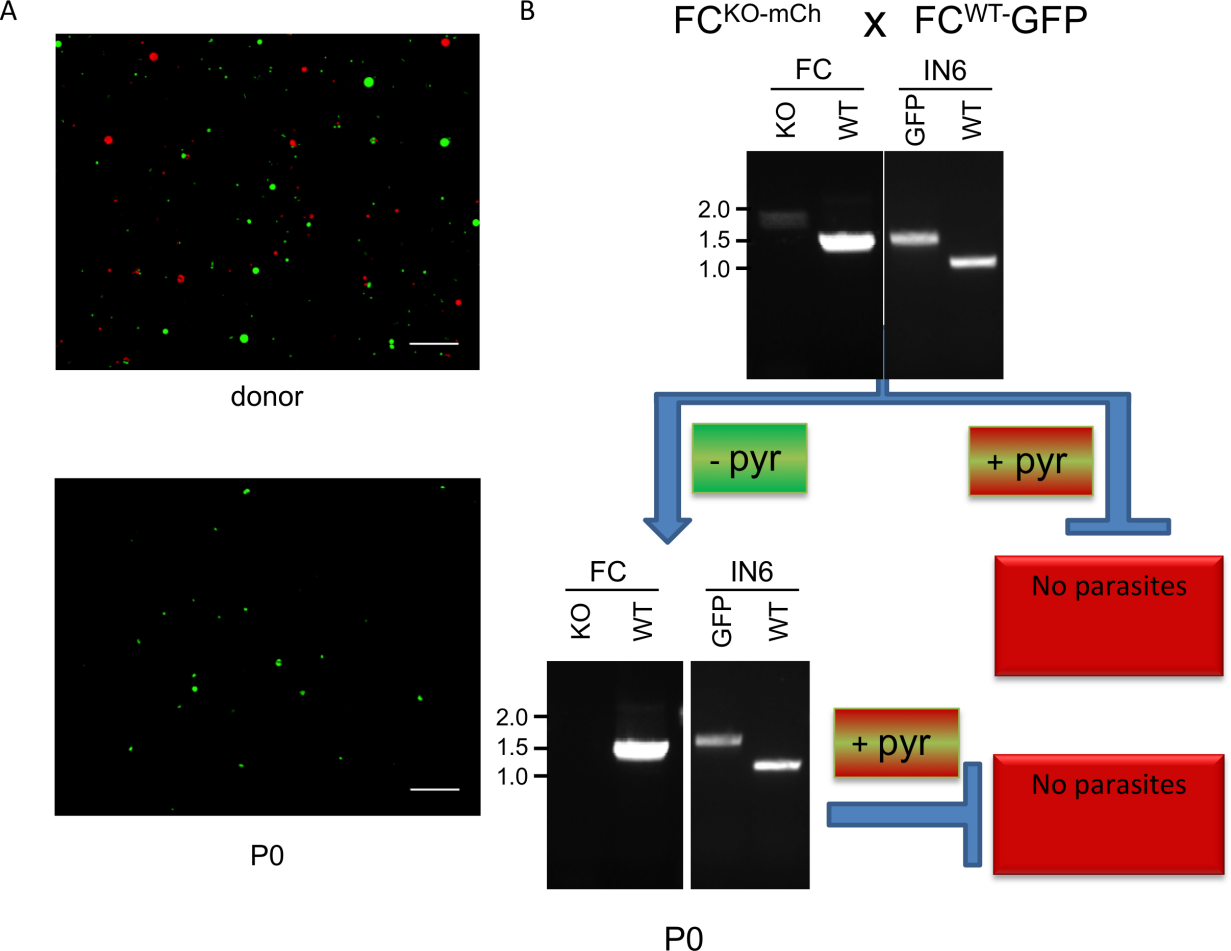
FC deficient parasites cannot complete the liver stage *in vivo*. (A) Epi-fluorescent images comparing the expression of mCherry and GFP before and after transmission. Both GFP and mCherry are visible in parasites recovered from the blood of a donor mouse dual infected with FC^KOmCh^ and FC^WT^-GFP parasites (upper panel). In contrast only GFP expression is observed in a naïve mouse infected with the progeny (P0) of this cross (lower panel), confirming the essential nature of FC during liver stage development. scale bar: 100μm. B) Summary of the genotypes and pyrimethamine resistance phenotype following infection of naïve mice with sporozoites from the FC^KOmCh^ x FC^WT^-GFP cross. PCR screening detects parasites with both of the FC and GFP alleles in the donor mouse used to infect mosquitoes. The FC^KOmCh^ genotype cannot be detected in the blood of mice infected by parasites found in those mosquitoes, demonstrating its lethality in the liver stage. Pyrimethamine selection for parasites carrying the hDHFR inserted in the FC locus kills all parasites, confirming that FC^KOmCh^ sporozoites cannot infect naïve mice. FC^WT^-GFP parasites are susceptible to pyrimethamine treatment and are, therefore, not present after drug treatment.

To assay FC^KOmCh^ liver development *in vivo*, we used both bite-back and IV injection of purified salivary gland sporozoites to infect naïve mice. Positive PCR of FC^KOmCh^ parasites in mice livers 40–42 hours after IV injection of complemented sporozoites demonstrates that these parasites can successfully infect mouse hepatocytes *in vivo* (Fig 1F). To test if FC^KOmCh^ parasites could complete the liver stage in naïve mice, we then tested for blood stage parasites by microscopy and PCR. Blood stage patency was achieved in standard times frames from either mosquito bites or intravenous injection of sporozoites from each of our crosses, including those with FC^KOmCh^ parasites as one parent (Table 1). However, fluorescence microscopy revealed no mCherry expressing parasites in any of the infected mice (Fig 4A) indicating that parasites carrying FC^KOmCh^ could not complete the liver stage *in vivo*. All other expected gene combinations following recombination were detected by PCR, including the nek-4ko allele transmitted via the male FC^WT^-nek-4ko gamete, confirming that recombination had occurred (Figs 4B and S3).

To exclude the possibility that FC^KOmCh^ parasites were present at levels below the threshold of detection by microscopy or PCR, we applied pyrimethamine selection to the P0 mice infected with sporozoites generated by the FC^KOmCh^ x FC^WT-^GFP cross. The FC^WT-^GFP parasites lack pyrimethamine resistance, so drug pressure should selectively recover any FC^KOmCh^ parasites (in which *h*DHFR disrupts their FC gene) that had completed the liver stage. Following infection with sporozoites from the FC^KOmCh^ x FC^WT^-GFP cross, one group of mice was immediately treated with pyrimethamine. These mice remained free of parasites for 14 days after infection. A second group of mice were left untreated following sporozoite infection and then treated with pyrimethamine after the infection was established (7 days after infection). All parasites were cleared after four days of pyrimethamine treatment, and no recrudescence was detected. These experiments eliminate the possibility that a small number of FC^KOmCh^ parasites complete the liver stage but are being masked by a larger population of FC^WT^ parasites. This confirms that the deletion of FC is lethal during the exoerythrocytic stage of *P*. *berghei*. FC^KOmCh^ parasites can establish, but not complete, a liver stage infection and this prevents them from generating a blood stage infection.

## Discussion

We confirmed that *P*. *berghei* rodent malaria parasites with a disrupted FC gene are viable during blood stage, can infect mosquitos and produce oocysts, but that these oocysts fail to produce sporozoites. As with many other mutants with their first observable effect in the mosquito stage, the absolute block in sporozoite production by heme pathway deletion mutants essentially prevents any subsequent assessment of the viability of these mutants in the sporozoite and liver stages. Our aim was to create a simple genetic system to complement the deletion of any mosquito stage essential gene using a heme enzyme as a test case. It has been a common strategy in *Plasmodium* parasites that can be transmitted in the lab, to complement mosquito stage defects by crossing mutant parasites to wild type parasites (S3 Table). This simple technique takes advantage of the heterokaryotic, polyploid nature of the mosquito stages to determine if the deleted gene is essential for transmission. However, this approach does not allow phenotypic analysis in the early stages of the mammalian infection because it yields a mixed population of wild type and mutants parasites that are indistinguishable from each other. By inserting a fluorescent marker into the deleted locus, we can definitively identify mutant parasites following recombination, thus enabling phenotypic characterization of these parasites during liver stages. Using this technique, we analysed the late mosquito, and early mammalian stage phenotype of the final enzyme in heme synthesis, FC.

We were able to generate haploid FC^KO^ sporozoites (Fig 1F), and (in agreement with findings for parasites lacking ALAS but chemically complemented with d-ALA) these FC^KOmCh^ sporozoites showed no significant defects in motility (S2 Fig, S2 Table) and could infect liver cells both *in vivo* and *in vitro* (Figs 1F and 3). ALAS is the first enzyme in the heme synthesis pathway and FC is the last, so it is reasonable to assume that the entire synthesis pathway is not essential for sporozoites. We note, however, that all sporozoites from the forced outcross to a colourless, female sterile parasite line (FC^WT^-nek-4ko) contained mCherry, even though the genotyping confirmed the presence of sporozoites carrying the non-fluorescent FC^WT^ locus (Fig 1F). This suggested significant carryover of mRNA, protein and/or heme from the cytoplasm of the multinucleate oocyst that could transiently complement FC^KOmCh^ sporozoites. By crossing parasites expressing different fluorophores from the same genetic locus we confirmed that sporozoites can contain proteins expressed in the oocyst even when they lack the gene encoding these proteins (Fig 2). Therefore, the FC^KOmCh^ sporozoite phenotypic data must be interpreted with caution until further studies clarify the nature and persistence of the products carried over from the shared oocyst cytoplasm to the haploid sporozoite.

Parasites in mosquito and hepatic cells are postulated to have fewer host cell resources available to them in comparison to parasites in the erythrocytic environment [14]. Indeed, mitochondrial chemiosmotic respiration is significantly increased as parasites transition to the non-red blood cell stages, reflecting the activation of oxidative phosporyhylation to generate ATP [30]. Thus, it is not surprising that this stage has high demands for heme cores for cytochromes and that *de novo* heme biosynthesis is essential during the protracted oocyst stage of the *Plasmodium* life cycle, [5, 12, 13, 15]. Supplementing ALAS deficient parasites by exogenously applying the product of this enzyme during the mosquito stages has proven an elegant method to look at the role of the initial heme synthesis enzyme in the liver stage of parasite development [12, 15]. However, the complexity of the *Plasmodium* heme biosynthetic pathway, particularly being spread over three cellular compartments, suggests that the parasite may have differential abilities to compensate for loss of heme pathway components.

In our system, the specific phenotype of FC deficient parasites could be assessed throughout the entirety of liver stage development. Reassuringly, at the first phenotypic assessment at 24 hours, parasites could be definitively classified based on the expression of mCherry, suggesting there is no detectable carry over of fluorphores, and presumably other proteins, from the sporozoite at this early liver stage. It remains possible that very early liver stage development could be affected by residual protein from the oocyst, although the absence of any oocyst derived fluorphores at 24 hours post infection suggest a very limited contribution of oocyst derived products to overall development in the liver stage. mCherry expressing FC knockout parasites were present throughout the life cycle up to 68 hpi, but these had significant growth defects that arrest their progress in the middle of liver stage development. The reduced expression of MSP-1 (Fig 3D) further suggests that FC^KOmCh^ parasites are significantly impaired in the ability to develop normally beyond ~50 hours after liver cell invasion, the point at which MSP-1 is detectable [25]. This developmental block was confirmed by the slower growth rate (Fig 3A and 3B), reduced number of nuclear division (Fig 3C) and inability to complete development, as demonstrated by the inability to produce merosomes (Table 2).

*In vivo* transmission confirmed the *in vitro* liver stage phenotype of FC^KOmCh^ parasites. Mutant parasites could be detected in the mouse liver 40–42 hours after infection with sporozoites (Fig 1F) but could not be detected in the subsequent blood stage infection by fluorescence or PCR, even when mice were infected by 10-fold more sporozoites than are required to generate 100% infection rates in the naïve mouse strain tested (Figs 4B and S3, Table 1). Recovery of all other combinations possible from sexual recombination confirmed successful crossing and infection of the naïve mice (Figs 4B and S3). The ability to specifically select for FC^KOmCh^ parasites using pyrimethamine treatment provided a further tool to assay for parasites carrying the FC^KOmCh^ locus, ensuring that no population of mutant parasites was present at levels below the sensitivity of our PCR screen and masked or overgrown by FC^WT^ parasites during subsequent blood stage infection. These screens also support the specific link between the observed phenotype and the FC locus. Rigorous, discipline-standard experimental technique in the development of the FC^KOmCh^ parasite line strongly supports a single, specific integration in this parasite line. However, the strict correlation observed between fluorescence, drug resistance and the FC gene deletion following sexual recombination and drug selection confirms that the observed phenotype is specifically caused by deletion of the FC locus.

Deletion of FC causes a different defect in parasite liver stage growth both *in vitro* and *in vivo* than that observed for parasites lacking the upstream heme pathway enzyme ALAS [15]. Although it was initially reported that ALAS activity is essential to generate infectious sporozoites [12], a subsequent study found that *P*. *berghei* parasites can partially compensate for the loss of ALAS activity [15] allowing them to maintain limited infectivity *in vivo* [15]. An *in vitro* analysis showed that these parasites can complete the liver stage and produce a limited number of merosomes in the absence of ALAS activity [15] presumably by scavenging sufficient d-ALA from the host cell to compensate for the loss of this enzyme. In contrast, our data suggest such scavenging cannot compensate for the lack of FC activity. The *in vitro* liver stage phenotype observed for FC^KOmCh^ parasites is far more severe than that for parasites lacking ALAS and no merosome production was observed. This agrees with the *in vivo* findings that FC^KOmCh^ parasites can infect the liver but not complete this life stage.

The marked difference between knockouts of the first enzyme in the synthesis pathway and the last, likely relates to differences in the stability of the two products and their localization within the host cell. Heme is a highly reactive molecule that can be a significant generator of oxygen radicals [31], whereas d-ALA is stable enough to survive prolonged periods in culture or in sucrose solution fed to mosquitoes. After d-ALA formation by ALAS in the host mitochondrion, this substrate is transported to the host cell cytoplasm where ALAD, the next enzyme in the pathway is located. This makes it much more likely that salvageable levels of d-ALA are available in the host liver cell cytoplasm. In contrast, in a hepatic cell FC, and the heme it produces, are located in the mitochondrion. This organellar localization is likely to restrict the free diffusion of reactive heme into the liver cell cytoplasm, where the parasite is located, thus preventing the parasite from salvaging enough heme to compensate for a loss of the FC enzyme. By contrast, red blood cells lack any internal cellular structure, so parasites are surrounded by all the enzymes of the heme pathway and by copious amounts of cytosolic haemoglobin. Parasites are also actively degrading haemoglobin in their food vacuole, making more heme available and apparently rendering FC dispensable at this stage [12, 13, 16].

The essential nature of FC during the *P*. *berghei* liver stages described here completes the picture of heme requirements, either endogenous or scavenged, for survival across all stages of the parasite life cycle. It also contrasts the differing ability of the parasite to scavenge host resources to compensate for a loss of enzymes in the *de novo* synthesis pathway. In the red blood cell, the abundance of freely accessible heme, heme pathway intermediates and host cell enzymes allows the parasite to rely on scavenging to meet its heme needs. Host resources appear less available or less accessible during the mosquito stages, making a loss of any heme-synthesizing enzyme during the insect stage lethal. Sporozoites apparently do not require heme synthesis, perhaps having sufficient heme on board after sporogony to underpin their needs. Alternatively, sporozoites would have to scavenge for their heme requirements in the mosquito hemocoel, vertebrate skin, blood capillaries and liver. The liver stage appears more complicated, with the parasites able to partially compensate for the loss of ALAS by scavenging d-ALA from the host cytoplasm. However, in the absence of FC parasites are unable to access sufficient quantities of host heme (or FC) to survive this stage. This suggests that targeting heme synthesis with prophylactic drugs in the parasite and host cells is still possible, but must be carefully considered, given the ability of the parasite to compensate for the loss of certain steps in the pathway. In contrast, the phenotype of parasites lacking FC suggests that heme synthesis may not be an ideal pathway to target for genetic attenuation vaccine strategies, which are more effective the longer the parasite grows in the liver [32].

Despite its importance in vaccine development and prophylaxis, hepatic growth of malaria parasites remains one of the least explored areas of parasite biology. Reverse genetic approaches to liver stage analysis have been hampered by two aspects of *Plasmodium* molecular genetics: only blood stages can be genetically modified, and getting from the blood stage to the liver stages means that these genetically modified parasites must traverse the entire life cycle. Thus, analysis of liver stage phenotypes was compromised by the difficulty in generating infectious sporozoites in mutant parasites with lethal phenotypes at earlier life stages. The complementation strategy outlined here presents a straightforward, simple to implement, and widely applicable approach to extending the analysis window for the burgeoning list of gene mutations (S3 Table) that show their first effects during the mosquito stages. Thus, complementation provides a powerful tool to dissect the biology of the parasite during its earliest, most vulnerable stage in the mammalian host.

## Supporting information

S1 Text Supplementary methods. (DOCX)

S1 Fig Generation of FC^KOmCh^ and FC^WT^-GFP and PCR genotyping strategy. A) FC^KOmCh^ B) GFP in intergenic region of chromosome 6 C) nek4-ko [18].
(TIF)

S2 Fig Motility of FC^KOmCh^ salivary gland sporozoites. (ns; not significant; P>.05, χ^2^ test); A) percentage of motile sporozoites observed B) representative image of a motile sporozoites and trails labelled with anti-CSP/AlexaFluor 488 antibodies (green). scale bar: 10μm.
(TIF)

S3 Fig PCR Genotyping of donor parasites and P0 parasites generated by crossing FC^KOmCh^ nek-4wt x FC^WT^-mCh-nek-4ko. (TIF)

S1 Table List of PCR primers and predicted product sizes. (DOCX)

S2 Table Motility of FC^KOmCh^ salivary gland sporozoites. (DOCX)

S3 Table Partial list of *Plasmodium* spp. pathways/genes shown to be essential for mosquito stage development that could be candidates for analysis with the described genetic complementation strategy. (DOCX)
