# Supplementary material for: A novel genetic technique in Plasmodium berghei allows liver stage analysis of genes required for mosquito stage development and demonstrates that de novo heme synthesis is essential for liver stage development in the malaria parasite
Source: PLoS Pathog. 2017 Jun 15;13(6):e1006396. doi: 10.1371/journal.ppat.1006396 (PMC5472305; doi:10.1371/journal.ppat.1006396)
Supplement: S1 Text — (DOCX) [file ppat.1006396.s001.docx]

Supplementary Methods

Generation of pChSKLD plasmid

Human DHFR from pL0006 (MRA-775) and mCherry from pBAT [19] were amplified using primers D355-D296 and D356-D354 (Table S1), respectively. These primers added overlapping portions of the Viral 2A skip peptide [20] to each PCR product. The resultant fragments were used as the overlapping template in PCR with primers D356 and D296 to generate the mCherry-Skip-hDHFR insert that was cut with BspHI/XbaI and cloned into the XbaI/NcoI sites of PL0006 to create pLChSKD.

Generation of FCWT-GFP, FCWT-tdTomato, and FCWT-mCherry parasite lines.

Lines expressing GFP or mCherry in the absence of a selectable marker were created in a two-step process. 5’ and 3’ flanks of the intergenic region of chromosome 6 corresponding to those detailed in [19] were generated by PCR using primers D331-D332 and D326-D327 (Table S1), respectively and cloned into the EcoRI/NotI (5’) and ApaI/AflII (3’) sites of pDONR-P2/P3 (Invitrogen) to create the plasmid pINT65’3’P2-P3. The hDHR/yfcu positive-negative selectable marker cassette from pL0034 (MRA-849) was cloned into pINT65’3’P2-P3 using XmaI/PstI. The construct was linearized with NotI/AscI and transfected into Pb ANKA parasites as describe in Material and Methods. Transfectants were cloned and correct integration confirmed by PCR.

GFP flanked by the HSP70 [19] 5’ and 3’ UTRs in pbc-GFP@hsp70 (a kind gift of Robert Menard, Institute Pasteur) was inserted into the SacI/XmaI sites of pBluescript (Agilent) to create pBSKHG. The mCherry sequence was cut out of pLChSKD with AvrII/BamHI and cloned into the NheI/BglII site of pBSKHG, replacing the GFP to create pBSKHCh. The tdTomato sequence was amplified and cloned into pL0006 as mCherry for pLChSKD, cut with AvrII/BamHI and cloned into the NheI/BglII site of pBSKHG to create pBSKHTm. pBSKHG, pBSKHTm and pBSKHCh were cut with SacI/XmaI to liberate the HSP705’-FP-HSP703’ cassette, which was inserted into pINT65’3’P2-P3 to create pINT65’3’HG, pINT65’3’HTm, and pINT65’3’HCh. These plasmids were linearized and transfected as above, with selection on 5-FC, and cloned. Correct gene insertion was confirmed by PCR. All parasite lines were tested in the complete life-cycle to confirm previous findings that HSP70 5’ driven expression of GFP, tdTomato and mCherry in this locus does not impair parasite development [19]. The fidelity of all PCR amplified products was confirmed by sequencing.
