# Supplementary figures and images for: A novel genetic technique in Plasmodium berghei allows liver stage analysis of genes required for mosquito stage development and demonstrates that de novo heme synthesis is essential for liver stage development in the malaria parasite
Source: PLoS Pathog. 2017 Jun 15;13(6):e1006396. doi: 10.1371/journal.ppat.1006396 (PMC5472305; doi:10.1371/journal.ppat.1006396)

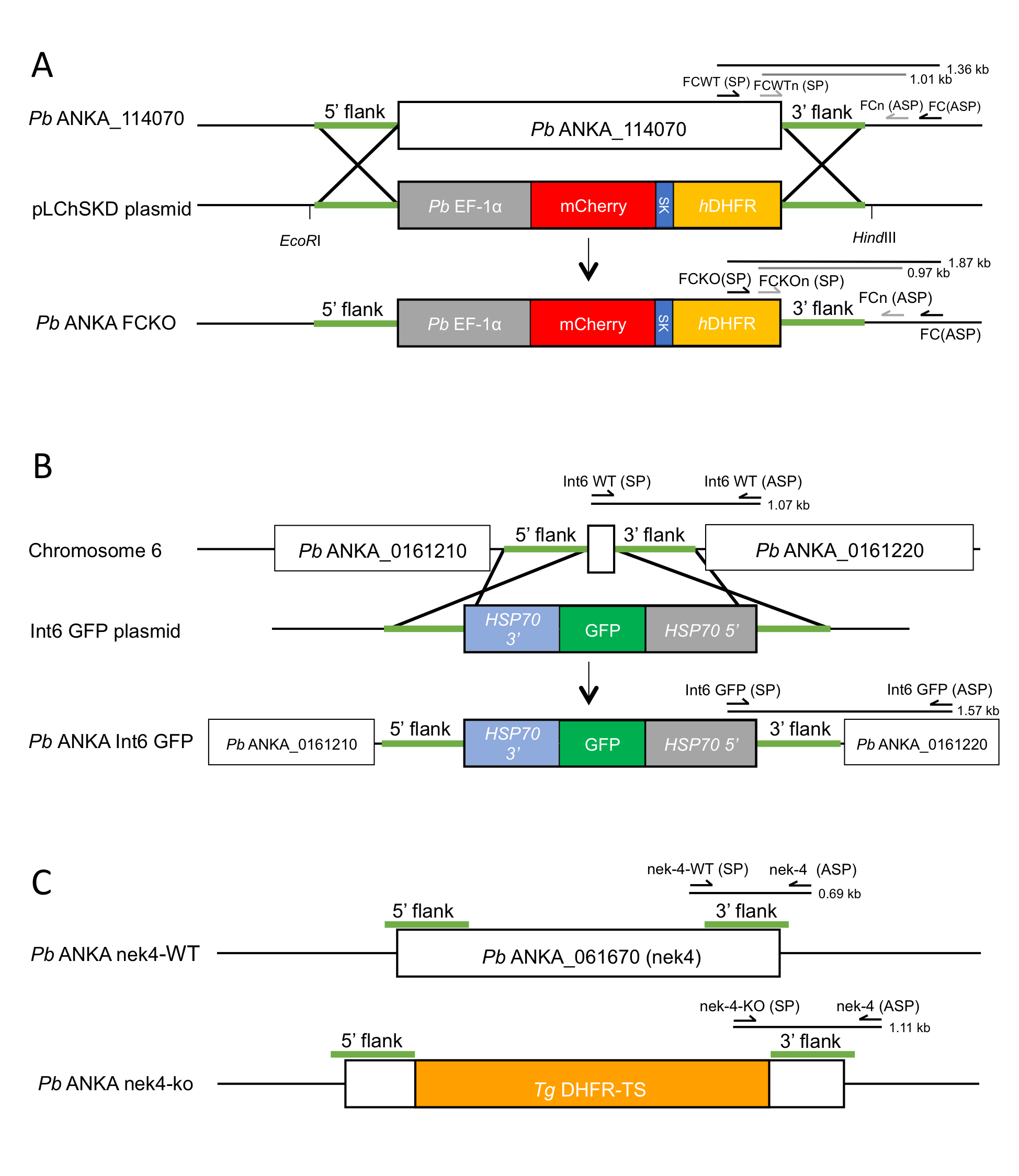

Supplement: S1 Fig — A) FCKOmCh B) GFP in intergenic region of chromosome 6 C) nek4-ko [18]. (TIF) [file ppat.1006396.s002.tif]

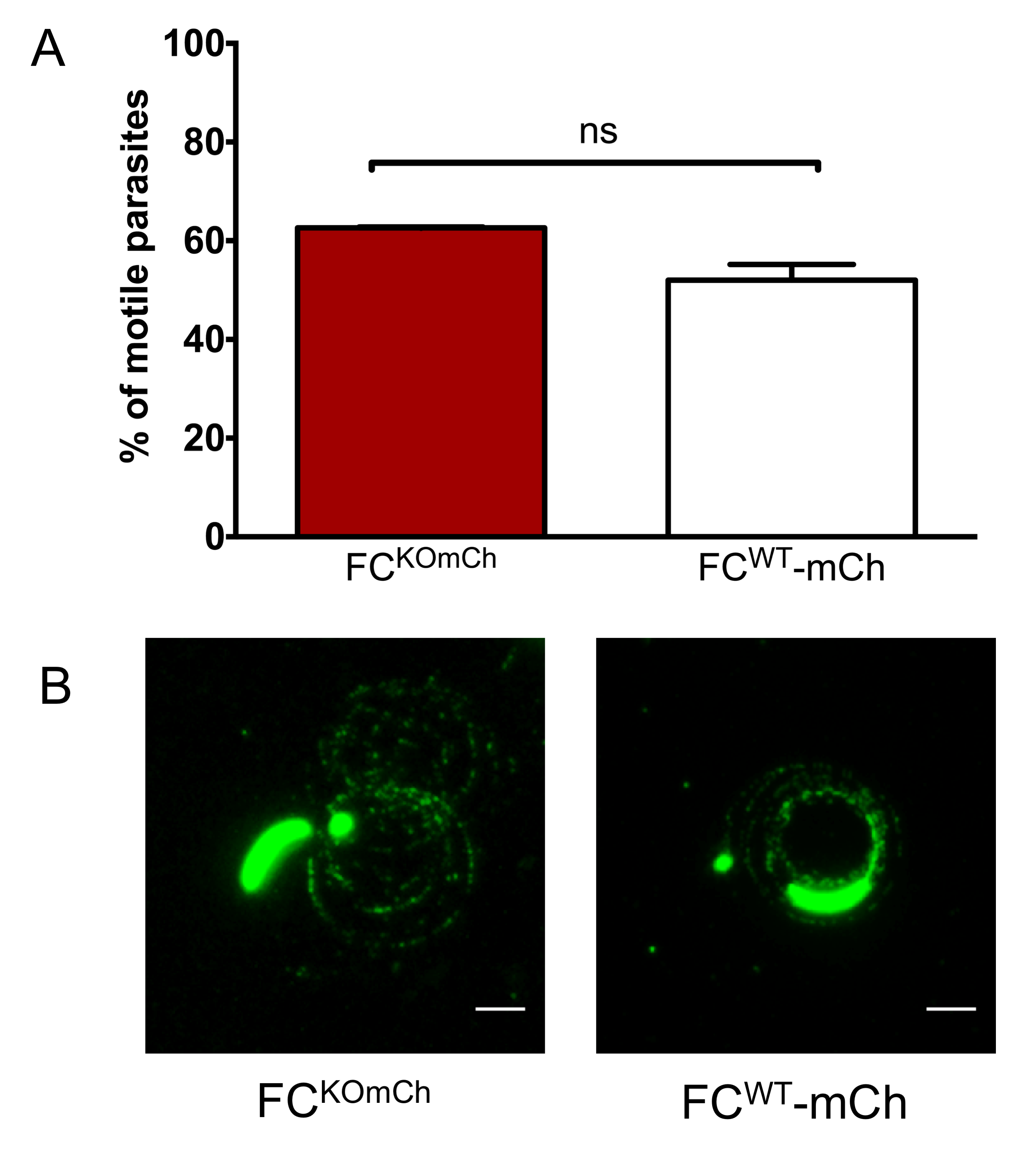

Supplement: S2 Fig — (ns; not significant; P>.05, χ2 test); A) percentage of motile sporozoites observed B) representative image of a motile sporozoites and trails labelled with anti-CSP/AlexaFluor 488 antibodies (green). scale bar: 10μm. (TIF) [file ppat.1006396.s003.tif]

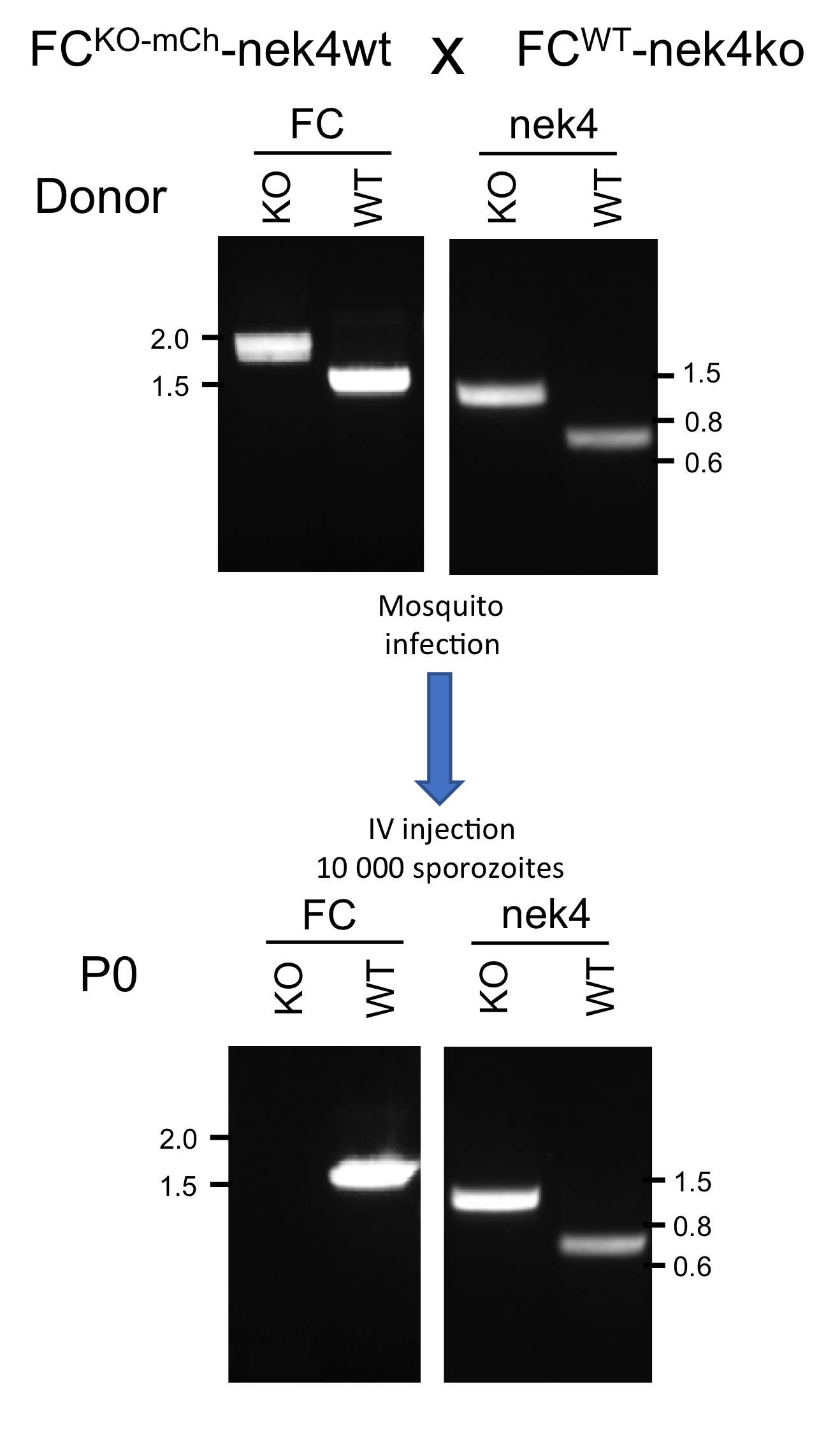

Supplement: S3 Fig — (TIF) [file ppat.1006396.s004.tif]
