# Supplementary material for: A novel genetic technique in Plasmodium berghei allows liver stage analysis of genes required for mosquito stage development and demonstrates that de novo heme synthesis is essential for liver stage development in the malaria parasite
Source: PLoS Pathog. 2017 Jun 15;13(6):e1006396. doi: 10.1371/journal.ppat.1006396 (PMC5472305; doi:10.1371/journal.ppat.1006396)
Supplement: S1 Table — (DOCX) [file ppat.1006396.s005.docx]

**Table S1. List of PCR primers.**

| **Primers** | **Primer Sequence (restriction sites in italics)** | | |
| --- | --- | --- | --- |
| **Vector Constrcution** | | | |
| **pLChSKD** | | | |
| D296 (ASP) | **5’***CCTAGG*CCATCATTCTTCTCATATACTTC**3’** | | |
| D354 (ASP) | **5’**TCCACGTCACCGATGTTAGAAGACTTCCTCTGCCCTCC  CTAGGCTTGTACAGCTCGTCCATG**3’** | | |
| D355 (SP) | **5’**AACATGCGGTGACGTGGAGGAGAATCCCGGCCCTGTC  GACGTTGGTTCGCTAAACTGCA**3’** | | |
| D356 (SP) | **5’***TCATGA*TCATGGTGAGCAAGGGCGAGGAG**3’** | | |
| **FC^WT^-GFP / FC^WT^-mCherry** | | | |
| D326 (SP) | **5’***GGGCCC*GGGTTCAGGAAACTTTTGTGTG**3’** | | |
| D327 (ASP) | **5’***CTTAAG*GCGCGCGCCATTCAGTTTGCCTATTTGC**3’** | | |
| D332 (SP) | **5’***GCGGCCGC*GTTTGTATTATGCACGCAAC**3’** | | |
| D333 (ASP) | **5’***GAATTCGGCGCGCC*TCATTTCCCCTGAATATAG**3’** | | |
| **Genotype Screening** | | | |
| **FCKO** | | | **Product Size (bp)** |
| *Pb* FCKO (SP) | | **5’**gaatcacccaggccatctta**3’** | 1868 |
| *Pb* FC (ASP) | | **5’**tgcatcatttttgtggcttg**3’** |  |
| *Pb* FCWT (SP) | | **5’**taatttgcccatccttttcg**3’** | 1362 |
| *Pb* FC (ASP) | | **5’**tgcatcatttttgtggcttg**3’** |  |
| *Pb* FCKOn (SP) | | **5’**tttttccttcaatttcgatgggtac**3’** | 965 |
| *Pb* FCn (ASP) | | **5’**accgagtaactggatcttcca**3’** |  |
| *Pb* FCWTn (SP) | | **5’**ttcaatcatatggaaatggacagtt**3’** | 1014 |
| *Pb* FCn (ASP) | | **5’**accgagtaactggatcttcca**3’** |  |
| **nek-4KO** | | |  |
| *Pb* nek-4 (SP) | | **5’**TCCAGATGGAGATGGCTGTC**3’** | 1112 |
| *Pb* nek-4 (ASP) | | **5’**CGAAGTTACATATTCAAATAGCCAAT**3’** |  |
| *Pb* nek-4 (SP) | | **5’**TTAGATCCCCGATTTACCAAA**3’** | 691 |
| *Pb* nek-4 (ASP) | | **5’**CGAAGTTACATATTCAAATAGCCAAT**3’** |  |
| **GFP** | | |  |
| *Pb* Int6 GFP (SP) | | **5’**AACTGAAAAAGAGCGCAAATG**3’** | 1568 |
| *Pb* Int6 GFP(ASP) | | **5’**CCCAGAATTCCCATGAACTG**3’** |  |
| *Pb* Int6 WT (SP) | | **5’**GCGGCCGCGTTTGTATTATGCACGCAAC**3’** | 1074 |
| *Pb* Int6 WT (ASP) | | **5’**TCTTGAGGAACGGATAGACAC**3’** |  |
