# Supplementary material for: A novel genetic technique in Plasmodium berghei allows liver stage analysis of genes required for mosquito stage development and demonstrates that de novo heme synthesis is essential for liver stage development in the malaria parasite
Source: PLoS Pathog. 2017 Jun 15;13(6):e1006396. doi: 10.1371/journal.ppat.1006396 (PMC5472305; doi:10.1371/journal.ppat.1006396)
Supplement: S2 Table — (DOCX) [file ppat.1006396.s006.docx]

|  | **Trails** | | | | | |
| --- | --- | --- | --- | --- | --- | --- |
|  | **0** | **linear or 1/2** | **1** | **2-5** | **6-10** | **>10** |
| **FC^KO-mCh^** | 35+ 4 | 25+ 2 | 12+ 6 | 20+ 3 | 2 | 0 |
| **FC^WT^-mCh** | 53+ 10 | 13+ 9 | 9 | 23+ 2 | 7+ 2 | 4+ 2 |

**Table S2. Motility of salivary gland sporozoites**
