# Supplementary material for: A novel genetic technique in Plasmodium berghei allows liver stage analysis of genes required for mosquito stage development and demonstrates that de novo heme synthesis is essential for liver stage development in the malaria parasite
Source: PLoS Pathog. 2017 Jun 15;13(6):e1006396. doi: 10.1371/journal.ppat.1006396 (PMC5472305; doi:10.1371/journal.ppat.1006396)
Supplement: S3 Table — (DOCX) [file ppat.1006396.s007.docx]

**Table S3. Partial list of *Plasmodium* spp. pathways/genes shown to be essential for mosquito stage development that could be candidates for analysis with the described genetic complementation strategy.**

| Description | Cellular function | Reference |
| --- | --- | --- |
| ACO* | Ookinete conversion | [33] |
| Actin 2* | Ookinete to oocyst formation | [34] |
| Actin capping protein β subunit* | Oocyst formation | [35] |
| ALAS | Oocyst maturation | [12, 13, 15]  (*P. falciparum)* |
| Api ap2-sp | sporogony | [36] |
| Api ap2-sp2 | Sporoblast formation | [36] |
| Api ap2-sp3 | Salivary gland invasion | [36] |
| ATP synthase β subunit* | Oocyst formation | [5] |
| BCKDH | Oocyst maturation | [37] |
| Cap380 | Oocyst maturation | [38] |
| c-CAP | Sporozoite production | [39] |
| CDLK | Sporozoite maturation | [40] |
| CDPK3 | Ookinete motility, mosquito midgut invasion | [41, 42] |
| CelTOS | Ookinete midgut traversal | [43] |
| Chitinase | Hydrolysis of peritrophic memebrane during ookinete midgut traversal | [44, 45]  (*P. falciparum*) |
| CPO | Oocyst maturation | [14] (*P. falciparum*) |
| CRMP1,2 | Salivary gland invasion | [46] |
| CSP | Sporozoite development within oocyst and sporozoite attachment to hepatocytes | [47] |
| CTRP* | Oocyst formation | [48] |
| CYC3* | Oocyst development, maturation and sporozoite formation | [49] |
| ECP1 | Egress from midgut oocyst | [50] |
| FabB/F | Oocyst to sporozoite development | [51] (*P. falciparum*) |
| FabI | Oocyst to sporozoite development | [51] (*P. falciparum*) |
| GAK | Sporozoite development | [40] |
| GGCS | Oocyst maturation | [52] |
| GluS | Oocyst development | [53] |
| GR | Oocyst maturation | [54, 55] |
| Ik2 | Latency of sporozoites in salivary glands | [56] |
| IMC1a | Sporogony | [57] |
| KGDH | Oocyst formation | [58] (*P. falciparum*) |
| LAP1/CCp3/SR* | Oocyst maturation and sporozoite formation | [17, 59] |
| LAP2/CCp1* | Oocyst maturation and sporozoite formation | [17, 59] |
| LAP3/CCp5 | Oocyst maturation and sporozoite formation | [17, 59] |
| LAP4/CCp2* | Oocyst maturation and sporozoite formation | [17], [59] |
| LAP5* | Oocyst maturation and sporozoite formation | [17, 59, 60] |
| LAP6* | Oocyst maturation and sporozoite formation | [59] |
| MAEBL | Salivary gland invasion | [61] |
| MAOP | Midgut invasion | [62] |
| MDH* | Oocyst formation | [33] |
| MISFIT (formin-like protein)* | Oocyst development | [63] |
| Nek-2,4 | Ookinete genome replication | [18] |
| P25 | Ookinete surface protein; ookinete survival in the mosquito midgut, traversal of the epithelium; ookinete-oocyst transformation | [64] |
| P28 | Ookinete surface protein; ookinete survival in the mosquito midgut, traversal of the epithelium; ookinete-oocyst transformation | [64] |
| Palmityol transferase DHHC3* | Gliding motility in ookinetes and sporozoites | [65] |
| PANK1,2 | Optimal ookinete formation and oocyst development | [54, 66] |
| PBGD | Oocyst maturation | [14, 15, 67]  (*P. falciparum*) |
| PEPC* | Oocyst formation | [33] |
| PEPCK | Oocyst development | [53] |
| PK7 | Oocyst development and sporogony | [40] |
| POS1-10 | Ookinete surface protein | [68] |
| PPLP3-5 | Ookinete midgut traversal | [68, 69] |
| PPM5* | Oocyst development | [70] |
| PSOP 1,2,6,7,12 | Ookinete-secreted proteins | [68] |
| PSOP13* | Sporozoite development | [60] |
| PSOP25 | Ookinete maturation and formation of oocyst | [71] |
| S-acyl-transferase DHHC10* | Sporozoite formation | [72] |
| SDH | Oocyst formation | [73] |
| SHLP1 | Ookinete formation | [74] |
| SOAP | Ookinete midgut traversal | [75] |
| TRAP | Salivary gland invasion and infection of hepatocytes | [76] |
| TRP1 | Oocyst egress and salivary gland invasion | [77] |
| Type II NADH: Ubiquinone dehydrogenase* | Oocyst maturation | [78] |
| UROD | Oocyst maturation | [15, 67] |
| WARP | Ookinete midgut traversal | [79, 80] |

* Genes where knockouts have been complemented through the mosquito stage by genetic crossing but not analysed for phentoypes in the liver stage. All the genes listed here are performed with *P. berghei* unless indicated otherwise. ACO; Acotinase, ALAS; Aminolevulinic acid synthase, ApiAP2; AP2 DNA-binding domain, BCKDH; Branched-chain amino acid dehydrogenase Cap380; Capsule protein 380, c-CAP; Cyclase-associated protein, CDLK; CDPK-like kinase, CDPK; Calcium-dependent protein kinase, CelTOS; Cell-traversal protein for ookinete and sporozoites, CPO; Coproporphyrinogen oxidase, CRMP; Cysteine repeat modular protein, CSP; Circumsporozoite protein, CTRP; Circumsporozoite protein and thrombospondin-related adhesive protein [TRAP]-related protein, CYC3; P-type cyclin 3, ECP1; Egress cysteine protease I, FabB/F; β-ketoacyl-ACP synthase II, FabI; Enoyl-ACP reductase, GAK; Cyclin G-associated kinase, GGCS; Gamma-glutamylcystein synthetase, GluS; Glutamate synthase, GR; Glutathione reductase, IK2; Initiation factor-2α kinase, IMC1a; Inner membrane complex protein, KGDH; α-keto-glutarate dehydrogenase, LAP; LCCL/ lectin adhesive-like protein 1, MAEBL; Apical membrane antigen/erythrocyte binding like protein, MAOP; Membrane-attack ookinete protein, MDH; Malate dehydrogenase, Nek; NIMA-related kinase, PANK; Putative pantothenase Kinase, PBGD; Porphobilinogen deaminase, PEPC; Phosphoenolpyruvate carboxylase, PEPCK; Phosphoenolpyruvate carboxykinase, PK7; Protein kinase 7, POS; Putative ookinete surface-associated protein, PPLP; Plasmodial perforin-like protein, PPM; Metallo-dependent protein phosphatase, PSOP; Putative secreted ookinete proteins, SDH; Succinate dehydrogenase, SHLP; *Shewanellla*-like protein phosphatase, SOAP; Secreted ookinete adhesive protein, TRAP; Thrombosponin-related adhesive protein, TRP1; Thrombosponidin-related protein, UROD; Uroporphyrinogen decarboxylase, WARP; Von Willebrand factor A domain-related protein

**Supplementary References**

33. Srivastava A, Creek DJ, Evans KJ, De Souza D, Schofield L, Muller S, et al. Host reticulocytes provide metabolic reservoirs that can be exploited by malaria parasites. PLoS Pathog. 2015;11(6).

34. Andreadaki M, Morgan RN, Deligianni E, Kooij TWA, Santos JM, Spanos L, et al. Genetic crosses and complementation reveal essential functions for the *Plasmodium* stage-specific actin2 in sporogonic development. Cell Microbiol. 2014;16(5):751-67.

35. Ganter M, Schuler H, Matuschewski K. Vital role for the *Plasmodium* actin capping protein (CP) beta-subunit in motility of malaria sporozoites. Mol Microbiol. 2009;74(6):1356-67.

36. Modrzynska K, Pfander C, Chappell L, Yu L, Suarez C, Dundas K, et al. A Knockout screen of apiap2 genes reveals networks of interacting transcriptional regulators controlling the *Plasmodium* life cycle. Cell Host Microbe. 2017;21(1):11-22.

37. Oppenheim RD, Creek DJ, Macrae JI, Modrzynska KK, Pino P, Limenitakis J, et al. BCKDH: the missing link in apicomplexan mitochondrial metabolism is required for full virulence of *Toxoplasma gondii* and *Plasmodium berghei*. PLoS Pathog. 2014;10(7):e1004263.

38. Srinivasan P, Fujioka H, Jacobs-Lorena M. PbCap380, a novel oocyst capsule protein, is essential for malaria parasite survival in the mosquito. Cell Microbiol. 2008;10(6):1304-12.

39. Hliscs M, Sattler JM, Tempel W, Artz JD, Dong A, Hui R, et al. Structure and function of a G-actin sequestering protein with a vital role in malaria oocyst development inside the mosquito vector. J Biol Chem. 2010;285(15):11572-83.

40. Tewari R, Straschil U, Bateman A, Bohme U, Cherevach I, Gong P, et al. The systematic functional analysis of *Plasmodium* protein kinases identifies essential regulators of mosquito transmission. Cell Host Microbe. 2010;8(4):377-87.

41. Ishino T, Orito Y, Chinzei Y, Yuda M. A calcium-dependent protein kinase regulates *Plasmodium* ookinete access to the midgut epithelial cell. Mol Microbiol. 2006;59(4):1175-84.

42. Siden-Kiamos I, Ecker A, Nyback S, Louis C, Sinden RE, Billker O. *Plasmodium berghei* calcium-dependent protein kinase 3 is required for ookinete gliding motility and mosquito midgut invasion. Mol Microbiol. 2006;60(6):1355-63.

43. Kariu T, Ishino T, Yano K, Chinzei Y, Yuda M. CelTOS, a novel malarial protein that mediates transmission to mosquito and vertebrate hosts. Mol Microbiol. 2006;59(5):1369-79.

44. Dessens JT, Mendoza J, Claudianos C, Vinetz JM, Khater E, Hassard S, et al. Knockout of the rodent malaria parasite chitinase pbCHT1 reduces infectivity to mosquitoes. Infect Immun. 2001;69(6):4041-7.

45. Tsai YL, Hayward RE, Langer RC, Fidock DA, Vinetz JM. Disruption of *Plasmodium falciparum* chitinase markedly impairs parasite invasion of mosquito midgut. Infect Immun. 2001;69(6):4048-54.

46. Thompson J, Fernandez-Reyes D, Sharling L, Moore SG, Eling WM, Kyes SA, et al. *Plasmodium* cysteine repeat modular proteins 1-4: complex proteins with roles throughout the malaria parasite life cycle. Cell Microbiol. 2007;9(6):1466-80.

47. Menard R, Sultan AA, Cortes C, Altszuler R, vanDijk MR, Janse CJ, et al. Circumsporozoite protein is required for development of malaria sporozoites in mosquitoes. Nature. 1997;385(6614):336-40.

48. Yuda M, Sakaida H, Chinzei Y. Targeted disruption of the *Plasmodium berghei* CTRP gene reveals its essential role in malaria infection of the vector mosquito. J Exp Med. 1999;190(11):1711-5.

49. Roques M, Wall RJ, Douglass AP, Ramaprasad A, Ferguson DJ, Kaindama ML, et al. *Plasmodium* P-type cyclin CYC3 modulates endomitotic growth during oocyst development in mosquitoes. PLoS Pathog. 2015;11(11):e1005273.

50. Aly ASI, Matuschewski K. A malarial cysteine protease is necessary for *Plasmodium* sporozoite egress from oocysts. J Exp Med. 2005;202(2):225-30.

51. van Schaijk BCL, Kumar TRS, Vos MW, Richman A, van Gemert GJ, Li T, et al. Type II fatty acid biosynthesis is essential for *Plasmodium falciparum* sporozoite development in the midgut of *Anopheles* mosquitoes. Eukaryot Cell. 2014;13(5):550-9.

52. Vega-Rodriguez J, Franke-Fayard B, Dinglasan RR, Janse CJ, Pastrana-Mena R, Waters AP, et al. The glutathione biosynthetic pathway of *Plasmodium* is essential for mosquito transmission. PLoS Pathog. 2009;5(2):e1000302.

53. Srivastava A, Philip N, Hughes KR, Georgiou K, MacRae JI, Barrett MP, et al. Stage-specific changes in *Plasmodium* metabolism required for differentiation and adaptation to different host and vector environments. PLoS Pathog. 2016;12(12):e1006094.

54. Pastrana-Mena R, Dinglasan RR, Franke-Fayard B, Vega-Rodriguez J, Fuentes-Caraballo M, Baerga-Ortiz A, et al. Glutathione reductase-null malaria parasites have normal blood stage growth but arrest during development in the mosquito. J Biol Chem. 2010;285(35):27045-56.

55. Buchholz K, Putrianti ED, Rahlfs S, Schirmer RH, Becker K, Matuschewski K. Molecular genetics evidence for the *in vivo* roles of the two major NADPH-dependent disulfide reductases in the malaria parasite. J Biol Chem. 2010;285(48):37388-95.

56. Zhang M, Fennell C, Ranford-Cartwright L, Sakthivel R, Gueirard P, Meister S, et al. The *Plasmodium* eukaryotic initiation factor-2alpha kinase IK2 controls the latency of sporozoites in the mosquito salivary glands. J Exp Med. 2010;207(7):1465-74.

57. Khater EI, Sinden RE, Dessens JT. A malaria membrane skeletal protein is essential for normal morphogenesis, motility and infectivity of sporozoites. J Cell Biol. 2004;167(3):425-32.

58. Ke H, Lewis IA, Morrisey JM, McLean KJ, Ganesan SM, Painter HJ, et al. Genetic investigation of tricarboxylic acid metabolism during the *Plasmodium falciparum* life cycle. Cell Rep. 2015;11(1):164-74.

59. Raine JD, Ecker A, Mendoza J, Tewari R, Stanway RR, Sinden RE. Female inheritance of malarial lap genes is essential for mosquito transmission. PLoS Pathog. 2007;3(3):e30.

60. Ecker A, Bushell ES, Tewari R, Sinden RE. Reverse genetics screen identifies six proteins important for malaria development in the mosquito. Mol Microbiol. 2008;70(1):209-20.

61. Kariu T, Yuda M, Yano K, Chinzei Y. MAEBL is essential for malarial sporozoite infection of the mosquito salivary gland. J ExpMed. 2002;195(10):1317-23.

62. Kadota K, Ishino T, Matsuyama T, Chinzei Y, Yuda M. Essential role of membrane-attack protein in malarial transmission to mosquito host. Proc Nat Acad Sci USA. 2004;101(46):16310-5.

63. Bushell ESC, Ecker A, Schlegelmilch T, Goulding D, Dougan G, Sinden RE, et al. Paternal effect of the nuclear formin-like protein MISFIT on *Plasmodium* development in the mosquito vector. PLoS Pathog. 2009;5(8).

64. Tomas AM, Margos G, Dimopoulos G, van Lin LHM, de Koning-Ward TF, Sinha R, et al. P25 and P28 proteins of the malaria ookinete surface have multiple and partially redundant functions. Embo J. 2001;20(15):3975-83.

65. Hopp CS, Balaban AE, Bushell ES, Billker O, Rayner JC, Sinnis P. Palmitoyl transferases have critical roles in the development of mosquito and liver stages of *Plasmodium*. Cell Microbiol. 2016;18(11):1625-41.

66. Hart RJ, Cornillot E, Abraham A, Molina E, Nation CS, Ben Mamoun C, et al. Genetic characterization of *Plasmodium* putative Pantothenate Kinase genes reveals their essential role in malaria parasite transmission to the mosquito. Sci Rep. 2016;6.

67. Posayapisit N, Songsungthong W, Koonyosying P, Falade MO, Uthaipibull C, Yuthavong Y, et al. Cytochrome c and c1 heme lyases are essential in *Plasmodium berghei*. Mol Biochem Parasitol. 2016;210(1-2):32-6.

68. Kaneko I, Iwanaga S, Kato T, Kobayashi I, Yuda M. Genome-wide identification of the target genes of AP2-O, a *Plasmodium* AP2-family transcription factor. PLoS Pathog. 2015;11(5):e1004905.

69. Wirth CC, Bennink S, Scheuermayer M, Fischer R, Pradel G. Perforin-like protein PPLP4 is crucial for mosquito midgut infection by *Plasmodium falciparum*. Mol Biochem Parasitol. 2015;201(2):90-9.

70. Guttery DS, Poulin B, Ramaprasad A, Wall RJ, Ferguson DJ, Brady D, et al. Genome-wide functional analysis of *Plasmodium* protein phosphatases reveals key regulators of parasite development and differentiation. Cell Host Microbe. 2014;16(1):128-40.

71. Zheng W, Liu F, He Y, Liu Q, Humphreys GB, Tsuboi T, et al. Functional characterization of *Plasmodium berghei* PSOP25 during ookinete development and as a malaria transmission-blocking vaccine candidate. Parasit Vectors. 2017;10(1):8.

72. Santos JM, Duarte N, Kehrer J, Ramesar J, Avramut MC, Koster AJ, et al. Maternally supplied S-acyl-transferase is required for crystalloid organelle formation and transmission of the malaria parasite. Proc Natl Acad Sci USA. 2016;113(26):7183-8.

73. Hino A, Hirai M, Tanaka TQ, Watanabe Y, Matsuoka H, Kita K. Critical roles of the mitochondrial complex II in oocyst formation of rodent malaria parasite *Plasmodium berghei*. J Biochem. 2012;152(3):259-68.

74. Patzewitz EM, Guttery DS, Poulin B, Ramakrishnan C, Ferguson DJP, Wall RJ, et al. An ancient protein phosphatase, SHLP1, is critical to microneme development in *Plasmodium* ookinetes and parasite transmission. Cell Rep. 2013;3(3):622-9.

75. Dessens JT, Siden-Kiamos I, Mendoza J, Mahairaki V, Khater E, Vlachou D, et al. SOAP, a novel malaria ookinete protein involved in mosquito midgut invasion and oocyst development. Mol Microbiol. 2003;49(2):319-29.

76. Mueller AK, Kohlhepp F, Hammerschmidt C, Michel K. Invasion of mosquito salivary glands by malaria parasites: Prerequisites and defense strategies. Int J Parasitol. 2010;40(11):1229-35.

77. Klug D, Frischknecht F. Motility precedes egress of malaria parasites from oocysts. Elife. 2017;6.

78. Boysen KE, Matuschewski K. Arrested oocyst maturation in *Plasmodium* parasites lacking type II NADH:ubiquinone dehydrogenase. J Biol Chem. 2011;286(37):32661-71.

79. Li FW, Templeton TJ, Popov V, Comer JE, Tsuboi T, Torii M, et al. *Plasmodium* ookinete-secreted proteins secreted through a common micronemal pathway are targets of blocking malaria transmission. J Biol Chem. 2004;279(25):26635-44.

80. Yuda M, Yano K, Tsuboi T, Torii M, Chinzei Y. von Willebrand Factor A Domain-related Protein, a novel microneme protein of the malaria ookinete highly conserved throughout *Plasmodium* parasites. Mol Biochem Parasitol. 2001;116(1): 65-72.
